# Mild hypothermia modulates the expression of nestin and caspase-3 in the sub-granular zone and improves neurological outcomes in rats with ischemic stroke

**DOI:** 10.18632/oncotarget.22647

**Published:** 2017-11-24

**Authors:** Dan Yu, Xueying Wang, Feng Zhou, Liang Wang, Guoshuai Yang, Wei Zhong, Ying Li, Zhiping Zhou, Aiyue Wang, Yanhui Zhou

**Affiliations:** ^1^ Department of Neurology, Haikou Municipal Hospital, Xiangya Medical College, Central South University, Haikou 570208, Hainan, P. R. China; ^2^ Department of Neurology, Affiliated Hospital, Chifeng College, Chifeng 024005, Inner Mongolia, P. R. China

**Keywords:** mild hypothermia, nestin, caspase-3, sub-granular zone, cerebral ischemia/reperfusion injury

## Abstract

We assessed neurological outcomes, infarct volume, and the expression of nestin and caspase-3 in the hippocampal dentate gyrus following middle cerebral artery occlusion (MCAO) followed by reperfusion, with mild hypothermia (MH) treatment at the onset of ischemia in a MCAO rat model. Reperfusion began 2 hours after the MCAO model was set-up. MH treatment began at the onset of ischemia and was maintained for 4 hours. We evaluated neurological deficit score, brain infarct volumes, along with the immunohistochemical staining of nestin and caspase-3 in the sub-granular zone of the injured hemisphere on the 1st, 3rd, 7th, and 14th day after the onset of ischemia. Correlations between the number of nestin-positive (nestin^+^) cells, caspase-3-positive (caspase-3^+^) cells with infarct volume, as well as neurological deficit scores, were evaluated by linear regression. MH significantly promoted survival, reduced mortality, improved neurological deficit score, reduced brain infarct volume, increased the number of neural stem/progenitor cells and inhibited neuronal apoptosis in the sub-granular zone of the injured hemisphere. The number of nestin^+^ cells correlated with neurological deficit score in the normothermic group, and with infarct volume in the hypothermia group except for the first day after the onset of ischemia. The number of caspase-3^+^ cells correlated with the neurological deficit score but not infarct volume. The neuroprotective effects of MH may be mediated by modulating neural stem/progenitor cells and neuronal apoptotic cells in the sub-granular zone of the injured hemisphere during cerebral ischemia/reperfusion injury.

## INTRODUCTION

Stroke is one of the most prominent causes of death and disability in adults; however, there are few treatments associated with this condition [1]. Focal cerebral ischemia represents ischemic stroke [2], and reperfusion injury represents a common complication of recanalization therapies following AIS [3]. Mild hypothermia (MH) is a promising neuroprotective therapy for stroke management. Thus far, a wide range of studies have determined that the neuroprotection offered by MH is pleiotropic in both the ischemic cascade and reperfusion injury [4]. However, the exact mechanism of neuroprotection provided by MH during acute ischemic stroke is not yet fully understood.

Nestin, a class VI intermediate filament protein, is expressed by neural stem cells and neural progenitor cells [5, 6]. These neural stem/progenitor cells have the potential to survive brain ischemia and participate in neurogenesis after stroke [7]. The number of nestin-immunopositive cells (nestin^+^ cells) is known to increase in the ischemic brain in response to focal cerebral ischemia/reperfusion injury [8, 9]. However, while the effects of MH upon nestin^+^ cells have been studied in neonatal cerebral hypoxic ischemic injury [10-13], as well as in adult global cerebral ischemia/reperfusion injury, which represents the clinical scenario of cardiac arrest [14-16], results have proved to be controversial and inconsistent. Furthermore, there have been no previous reports that have investigated the effect of MH on nestin^+^ cells during ischemia/reperfusion injury.

Caspase-3, a member of the family of cysteine proteases, is a major marker of neuronal apoptosis [17] in ischemic brain damage [18, 19]. MH exerts an inhibitory effect on neuronal apoptosis through caspase-3 mechanisms in the ischemic brain during adult focal cerebral ischemia/reperfusion injury [20-22]. However, studies investigating the effect of MH upon caspase-3 immunopositive cells (caspase-3^+^ cells) or neuronal apoptosis in the sub-granular zone remain sparse [10, 12].

In the current study, a rat model of ischemic stroke was created by middle cerebral artery occlusion (MCAO) followed by reperfusion (MCAO/R). MH started at the onset of ischemia and was maintained for 4 hours. We evaluated neurological outcomes, infarct volume, and the expression of nestin and caspase-3 in the sub-granular zone on the 1st, 3rd, 7th, and 14th day after the onset of ischemia. Survival and mortality rates were also determined at the same time points.

## RESULTS

### MH significantly promoted survival, reduced mortality, improved neurological function, and reduced brain infarct volume in rats with MCAO/R

**Figure 1:**
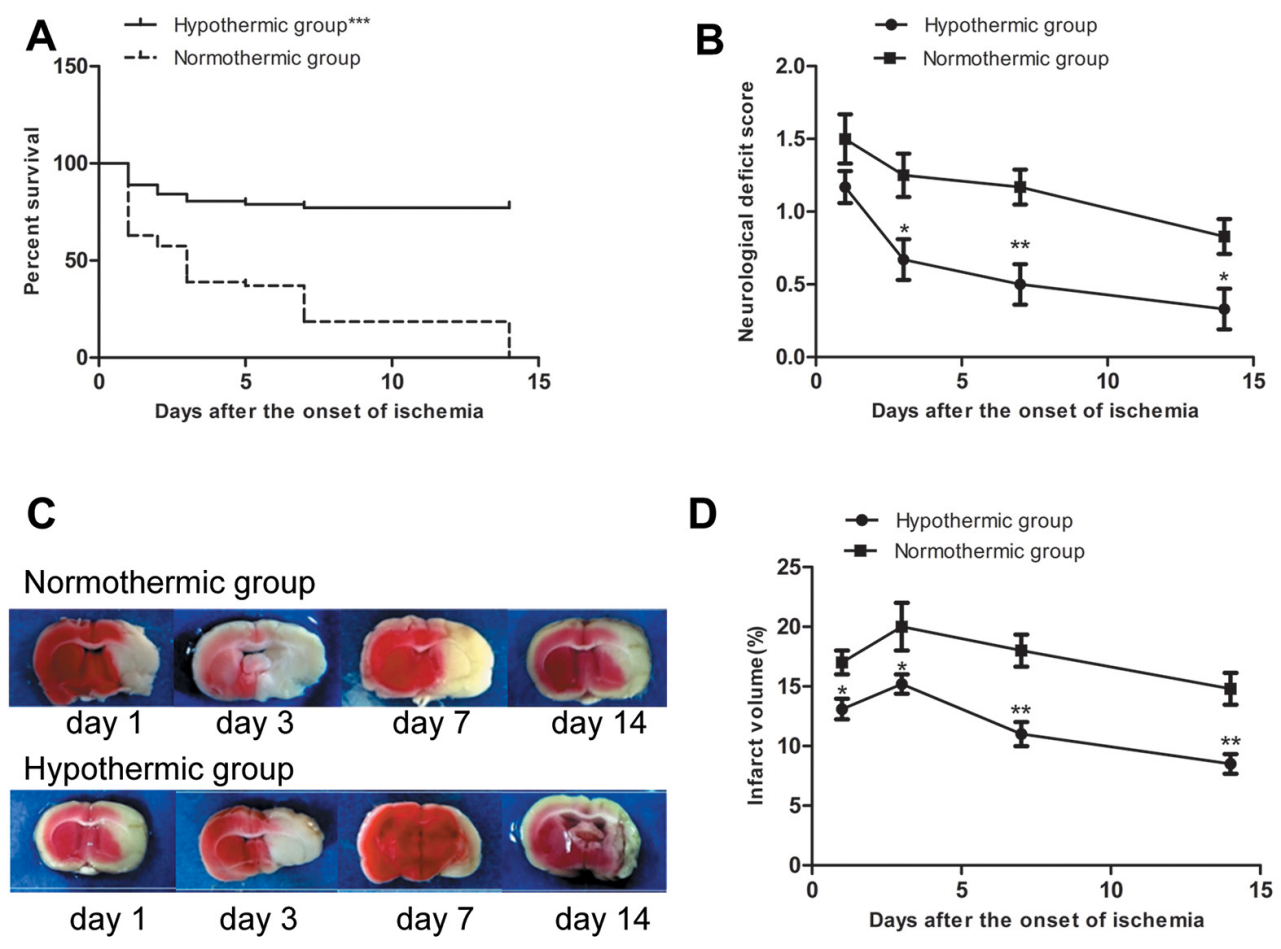
**(A)** Survival rate in normothermic and hypothermic groups. ^***^p < 0.001 compared with the normothermic group. **(B)** Neurological deficit score in normothermic and hypothermic groups. Compared with the normothermia group, ^*^p < 0.05 and ^**^p < 0.01. **(C)** Representative images of infarct areas that were stained with 2,3,5-triphenyltetrazolium chloride, where red tissues indicate normal tissue and white sections indicate infarction. **(D)** Proportion (%) of cerebral infarct volume in normothermia and hypothermia groups. Compared with normothermia group, ^*^p < 0.05, ^**^p < 0.01.

A total of 144 rats were used in our study. Twenty rats (10 each in the normothermic and hypothermic groups) died of subarachnoid hemorrhage, intracerebral hemorrhage, or anesthetic overdose, and therefore, were excluded from our study. Of the remaining 124 rats, all 16 rats in the sham group survived. Of the remaining 108 rats, 20 rats (16 in the normothermic group and 4 in the hypothermia group) died of extensive hemispheric infarction or severe cerebral ischemia were included in survival and mortality study between normothermic and hypothermic groups. There was a significant difference in survival rate between the normothermic and the hypothermic group when considered at the end of the experiment (Figure 1A). The total mortality rate in the hypothermia group was 7.4% (4/54 rats), which was significantly lower than the 29.6% (16/54 rats) rate in the normothermic group (χ^2^ = 8.84, ^***^p = 0.003 < 0.05).

The neurological deficit score based on a 5-point scale was used to estimate the neurological function. None of the rats shown neurological deficits prior to surgery. None of the rats in the sham group shown neurological deficits at any of the time points. Neurological deficit scores in the normothermic and hypothermic groups are shown in Figure 1B. None of the rats scored 0 on the 1st day after the onset of ischemia in either the normothermic or hypothermic groups. There was no significant difference between the two groups on the 1st day after the onset of ischemia. Compared to the normothermic group, MH significantly reduced the neurological deficit score on the 3rd, 7th, and 14th day after the onset of ischemia (^*^p < 0.05, ^**^p < 0.01).

TTC staining was used to visualize and quantify brain infarct volumes. Normal brain tissue appeared red, whereas infarct brain tissue appeared white. Rats in the sham group did not experience cerebral infarction. Representative images of infarct areas in normothermic and hypothermic rats are shown in Figure 1C. As shown in Figure 1D, compared to the normothermic group, MH significantly reduced the cerebral infarct size at each time point after the onset of ischemia (^*^p < 0.05, ^**^p < 0.01).

### MH significantly increased the number of nestin^+^ cells and reduced the number of caspase-3^+^ cells in the sub-granular zone of the injured hemisphere in rats with MCAO/R

**Figure 2:**
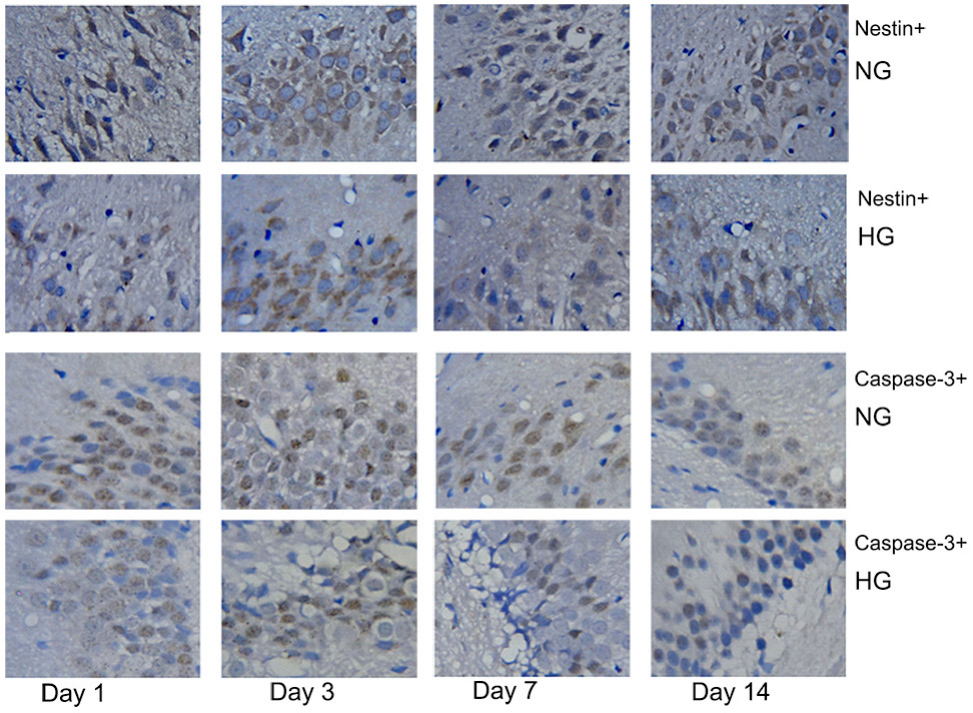
Rats in the normothermic and hypothermic groups had numerous nestin^+^ cells (brown-colored cytoplasm cells) and caspase-3^+^ cells (cells with a brown-colored nucleus) Original magnification × 400.

**Figure 3:**
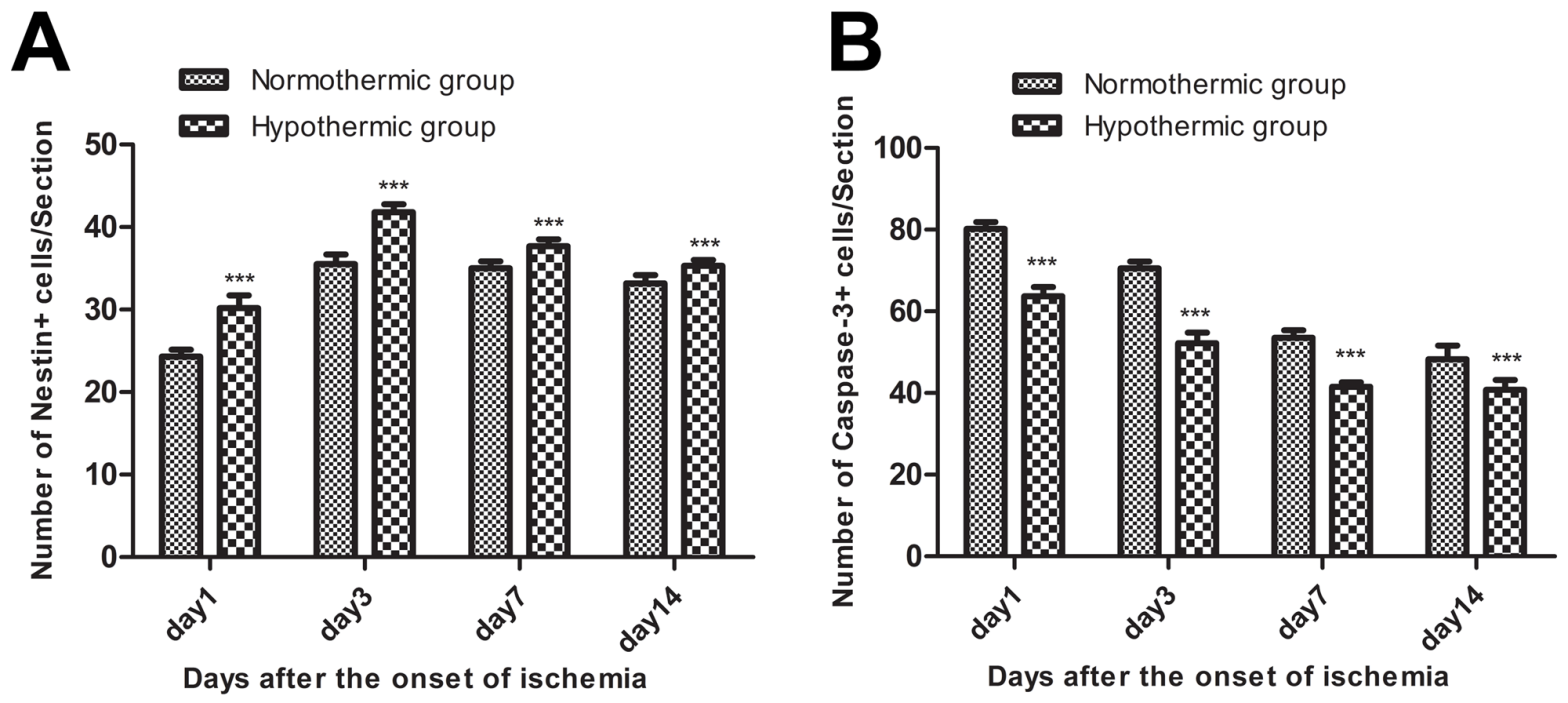
**(A)** Mild hypothermia significantly increased the number of nestin^+^ cells at all time points after the onset of ischemia. Compared with the normothermic group, ^***^p < 0.001. **(B)** Mild hypothermia significantly reduced the number of caspase-3^+^ cells at all time points after the onset of ischemia. Compared with the normothermic group, ^***^p < 0.001.

The immunohistochemical staining of nestin and caspase-3 was used to explore changes in neural stem/progenitor cells and neuronal apoptotic cells in the sub-granular zone of the injured hemisphere in rat with MCAO/R. No nestin^+^ cells or caspase-3^+^ cells were detected in rats from the sham group. However, numerous nestin+ cells and caspase-3^+^ cells were found in rats from the normothermic and hypothermic groups. Representative images of the immunopositive cells in normothermic and hypothermic rats are shown in Figure 2. Cells with nestin or caspase-3, which were seen to contain brown particles under a light microscope were considered as immunopositive cells. As shown in Figure 3A and Figure 3B, compared to the normothermic group, MH significantly increased the number of nestin^+^ cells and reduced the number of caspase-3^+^ cells at all time points after the onset of ischemia (^***^p < 0.05).

### Correlations between the number of nestin^+^ cells and caspase-3^+^ cells in the sub-granular zone of the injured hemisphere with infarct volume and neurological deficit score in rats with MCAO/R

**Figure 4:**
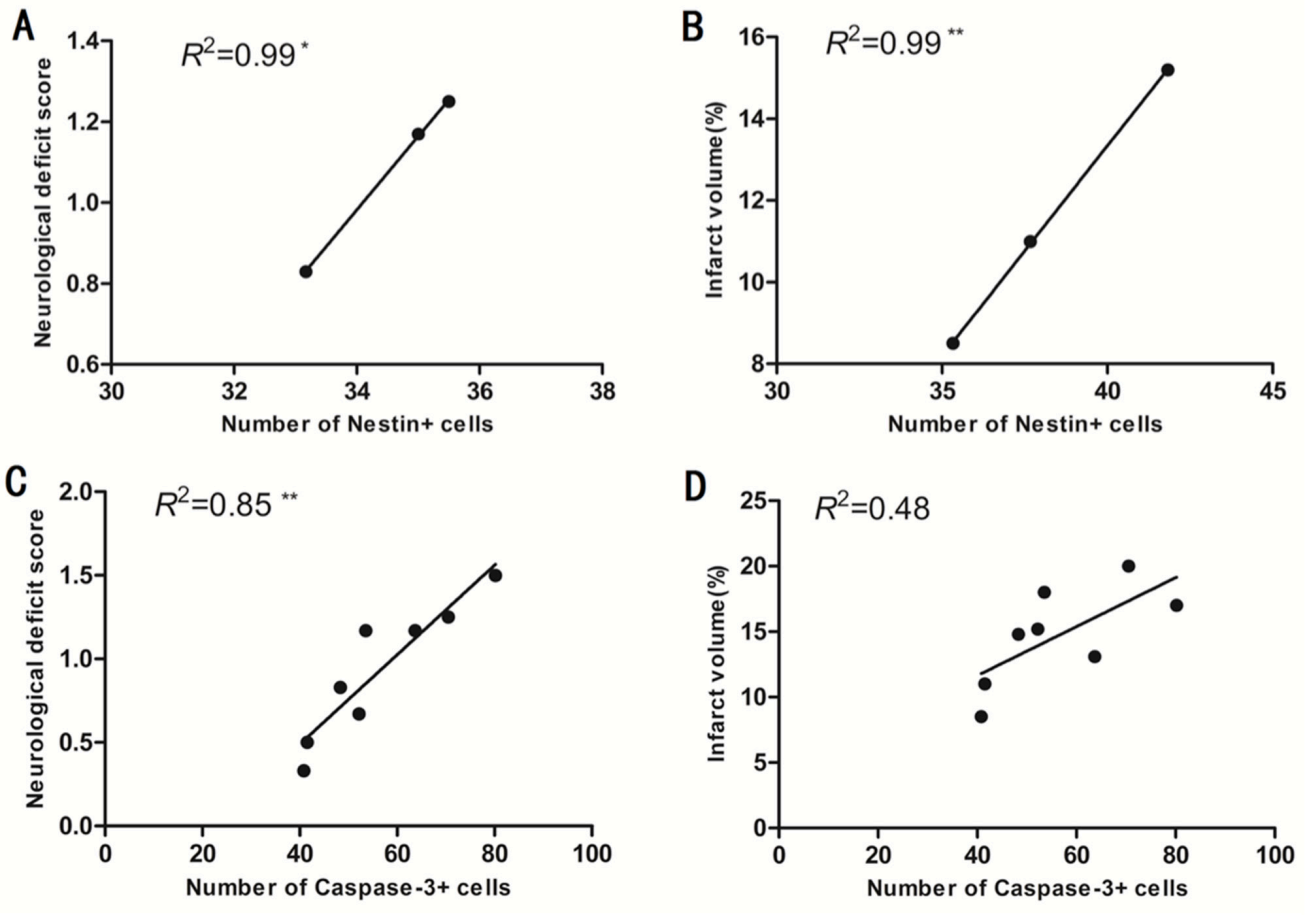
**(A)** The number of nestin^+^ cells in the sub-granular zone of the injured hemisphere correlated well with neurological deficit score in the normothermic rats when data from 1 day after the onset of ischemia was excluded from the analysis. ^*^p < 0.05. **(B)** The number of nestin^+^ cells in the sub-granular zone of injured hemisphere correlated with the percentage of cerebral infarct volume in hypothermic rats when data from day1 after the onset of ischemia was excluded. ^**^p < 0.01. **(C)** The number of caspase-3^+^ cells in the sub-granular zone of the injured hemisphere correlated well with the neurological deficit score in normothermic and hypothermic rats. ^**^p < 0.01. **(D)** The number of caspase-3^+^ cells in the sub-granular zone of the injured hemisphere did not correlate with the percentage of cerebral infarct volume in normothermic and hypothermic rats. P > 0.05.

We used linear regression and Pearson correlation coefficient analysis to determine correlations between the number of nestin^+^ cells and caspase-3^+^ cells in the sub-granular zone of the injured hemisphere with infarct volume and neurological deficit score in adult SD rats with MCAO/R. When considering pooled data from normothermic or hypothermic rats, and without considering the time points, the number of nestin^+^ cells in the sub-granular zone of the injured hemisphere correlated with neurological deficit score in the normothermic rats (^*^p < 0.05; Figure 4A), and correlated with cerebral infarct size in the hypothermic rats (^**^p < 0.01; Figure 4B) when data from the 1st day after the onset of ischemia was excluded. When considering pooled data from the normothermic and hypothermic rats, the number of caspase-3^+^ cells in the sub-granular zone of the injured hemisphere correlated with neurological deficit score (^**^p < 0.01; Figure 4C) but did not correlate with cerebral infarct size (p > 0.05; Figure 4D) in the normothermic and hypothermic rats.

## DISCUSSION

Therapeutic hypothermia is considered to improve survival in the case of global cerebral ischemia after cardiac arrest and perinatal asphyxia, but the efficacy of hypothermia in acute ischemic stroke remains relatively unstudied. Although there is very low-quality evidence to suggest against the routine induction of hypothermia as a means to improve survival in patients with acute ischemic stroke [23], MH is known to reduce mortality in different models of focal cerebral ischemia [24]. In animal models of MACO/R, MH improves survival and reduces mortality [25, 26]. In the current study, we found that when started at the onset of ischemia, MH significantly promoted survival and reduced mortality in adult SD rats with MCAO/R.

Until now, therapeutic hypothermia in patients with acute stroke has been applied only systematically by surface cooling, intravenous cooling, or cold saline infusions [27]. When considering the translation of this technique from an experimental situation to the clinic, there is already good evidence that MH, applied either during ischemia or during reperfusion, improves neurological function and reduces brain infarct volume; although data relating to systemic MH in MCAO/R rat models are limited [28-30]. Our results provide further evidence that systemic MH, started at the onset of ischemia, improves neurological function and reduces brain infarct volume in the rat MCAO/R. On the other hand, Karibe et al. [31] reported that systemic MH initiated 1 hour after the onset of ischemia had no effect on infarct volume in adult SD rats with MCAO/R.

We attempted to study the effect of MH on nestin^+^ cells in the sub-granular zone of the injured hemisphere in a MCAO/R rat model [10, 13]. Hypothermia treatment rescued nestin^+^ cells on the 1st day after injury in the sub-granular zone in a neonatal mouse with mild-to-moderate hypoxic-ischemic injury. MH did not change the number of nestin^+^ cells in the sub-granular zone and striatum on the 1st and 2nd week during the development of hypoxic-ischemic injury. Interestingly, we found that MH significantly increased the number of nestin^+^ cells in the sub-granular zone of the injured hemisphere at all time points after the onset of ischemia in rats with MCAO/R. As nestin is a major marker of neural stem/progenitor cells [6], and since neural stem/progenitor cells reside in the sub-granular zone, we considered that MH significantly increased the number of neural stem/progenitor cells in the sub-granular zone of the injured hemisphere in rats with MCAO/R. Our results are notably different from previous findings related to neonatal hypoxic-ischemic injury [10, 13]. Indeed, the pathophysiology of hypoxic-ischemic injury in neonates differs greatly from those of MCAO/R in adults. Although there are no previous reports investigating the effect of MH upon nestin^+^ cells in adult focal cerebral ischemia/reperfusion injury, MH has been reported to enhance endogenous neurogenesis in adult global cerebral ischemia/reperfusion injury [14, 16]. Soon after ischemic stroke, there is a proliferation and differentiation of neural stem/progenitor cells, which represents an important mechanism for neuronal restoration, however, such endogenous neurogenesis by itself is insufficient for effective brain repair after stroke as most newborn neurons die [32]. Thus, enhancing endogenous neurogenesis is a potential therapeutic strategy in stroke treatment [33].

MH exerts an inhibitory effect on brain cell apoptosis through caspase-3 mechanisms in rats with MCAO/R [20, 22]. We found that MH significantly reduced the number of caspase-3^+^ cells in the sub-granular zone of the injured hemisphere at all time points after the onset of ischemia in rats with MCAO/R. As caspase-3 is a major marker of neuronal apoptosis [17], we considered that MH significantly inhibited neuronal apoptosis in the sub-granular zone of the injured hemisphere in rats with MCAO/R. However, Sahin et al. [34] reported that MH had no effect on the immunohistochemical expression of caspase-3 in rats with MCAO/R. Our results are thus contradictory to the findings by Sahin et al., because they observed changes in the immunohistochemical expression of caspase-3 only at 24 hours after the onset of ischemia, and they used the MH in a range of 33-35°C, which was somewhat higher than used by ourselves. To the best of our knowledge, this study is the first to investigate the effect of MH on neuronal apoptosis in the sub-granular zone in adult focal cerebral ischemia/reperfusion injury.

We tried to address the possible associations between the number of nestin^+^ cells in the sub-granular zone of the injured hemisphere and infarct size and neurological deficit score in rats with MCAO/R. Surprisingly, we found that the number of nestin^+^ cells in the sub-granular zone of the injured hemisphere correlated well with neurological deficit score in the normothermic rats but with infarct volume in the hypothermic rats when data from the 1st day after the onset of ischemia was excluded. A few previous studies have demonstrated correlations between nestin and both infarct size and neurological deficit score on the 3rd, 7th or 14th day after the onset of ischemia in focal cerebral ischemia and/or reperfusion injury [35-37]. Our results thus confirm the correlation between nestin and neurological deficit score on the 3rd day after the onset of ischemia and extend this correlation up to the 14th day after the onset of ischemia in normothermic rats, suggesting that endogenous neural stem/progenitor cells in the sub-granular zone of the injured hemisphere are associated with focal cerebral ischemia/reperfusion injury. Such correlation in the normothermic rats may be the result of spontaneous endogenous neurogenesis induced by ischemia [16] and means that a higher neurological deficit score activates more endogenous neural stem/progenitor cells to be generated in the sub-granular zone of the injured hemisphere in adult focal cerebral ischemia/reperfusion injury. Disappointingly, this endogenous neurogenesis by itself is insufficient for effective brain repair after stroke as most newborn neurons do not survive, therefore stroke-induced neurological deficits are persistent. Conversely, the correlation between nestin and infarct volume on the 3rd, 7th or 14th day after the onset of ischemia was not observed in the normothermic group, but only existed in the hypothermic group. Such correlation found in the hypothermic group may be the result of enhanced endogenous neurogenesis induced by hypothermia [14, 16] and means that the enhanced endogenous neurogenesisin the sub-granular zone of the injured hemisphere produces more neural stem/progenitor cells to combat the larger infarct associated with focal cerebral ischemia/reperfusion injury.

Previous researchers [38, 39] have emphasized that there were significant correlations between caspase-3 and infarct volume and neurological deficit score at 24 hours after the onset of ischemia. We found that the number of caspase-3+ cells in the sub-granular zone of the injured hemisphere correlated well with neurological deficit score but not with infarct volume in adult SD rats with MCAO/R. We found that the number of caspase-3^+^ cells correlated well with neurological deficit score at 24 hours after the onset of ischemia. In contrast to previous findings [39, 40], we found that the number of caspase-3^+^ cells in the sub-granular zone of the injured hemisphere correlated well with neurological deficit score up to the 14th day after the onset of ischemia. This correlation suggests that the caspase-dependent neuronal apoptosis in the sub-granular zone of the injured hemisphere is clearly associated with adult focal cerebral ischemia/reperfusion injury and that MH exerts an inhibitory effect on brain cell apoptosis through caspase-3 mechanisms in the sub-granular zone of the injured hemisphere in adult focal cerebral ischemia/reperfusion injury. Therefore, stroke-induced neurological deficit scores can be reduced by MH. Additionally, in contrast to previous findings [39, 40], we found that there was no correlation between the number of caspase-3^+^ cells and infarct volume in our study. This is possibly because previous research studies investigated caspase-3 activity, plasma caspase-3 levels, or the number of caspase-3^+^ cells in the striatum, which were different parameters than those evaluated in our present study. Nevertheless, our data support the well-known concept that the caspase-dependent apoptotic pathway is involved in ischemic brain damage [18] and that MH exerts an inhibitory effect on brain cell apoptosis through caspase-3 mechanisms in adult focal cerebral ischemia/reperfusion injury.

This study has some limitations. First, we only measured nestin and not other markers of proliferation, migration or differentiation in neural stem/progenitor cells [41]. As a result, we were unable to verify how MH enhanced endogenous neurogenesis in the sub-granular zone of the injured hemisphere in a MCAO/R rat model. Second, our pooled analysis may have been affected by multiple factors [42]. Therefore, our correlations, particularly the unique correlations between the number of nestin^+^ cells in the sub-granular zone of the injured hemisphere with infarct volume and neurological deficit score in the MCAO/R model, require confirmation from future studies. Third, all rats were used randomly without specific selection, although focal cerebral ischemia/reperfusion injury was confirmed by infarct volume and neurological deficit score in rats with stroke, we cannot exclude the possibility of bias [43].

In conclusion, we identified that MH significantly promoted survival, reduced mortality, improved neurological function, reduced brain infarct volume, increased the number of neural stem/progenitor cells and inhibited neuronal apoptosis in the sub-granular zone of the injured hemisphere in a MCAO/R rat model. Furthermore, we found that there were significant correlations between the number of nestin^+^ cells and caspase^+^ cells in the sub-granular zone of the injured hemisphere with infarct volume and neurological deficit score in MCAO/R rats. Our findings provide new insight into the neuroprotective mechanisms associated with MH in focal cerebral ischemia/reperfusion injury and will be useful in the use of clinical hypothermia therapy for stroke patients. Further studies are now warranted to clarify why the number of nestin^+^ cells and caspase^+^ cells in the sub-granular zone of the injured hemisphere correlates with infarct volume and neurological deficit score in adult focal cerebral ischemia/reperfusion injury.

## MATERIALS AND METHODS

### Animal protocol

Adult male Sprague-Dawley (SD) rats, aged 12-16 weeks and weighing 250 -300g, were supplied by the Laboratory Animal Center of Central South University (Changsha, China). The rats were housed under standard husbandry conditions with free access to food and water. All experimental procedures were in accordance with the National Institute of Health (NIH) Guide for the Care and Use of Laboratory Animals. The study was approved by the Ethics Committee of Haikou Municipal Hospital.

**Figure 5:**
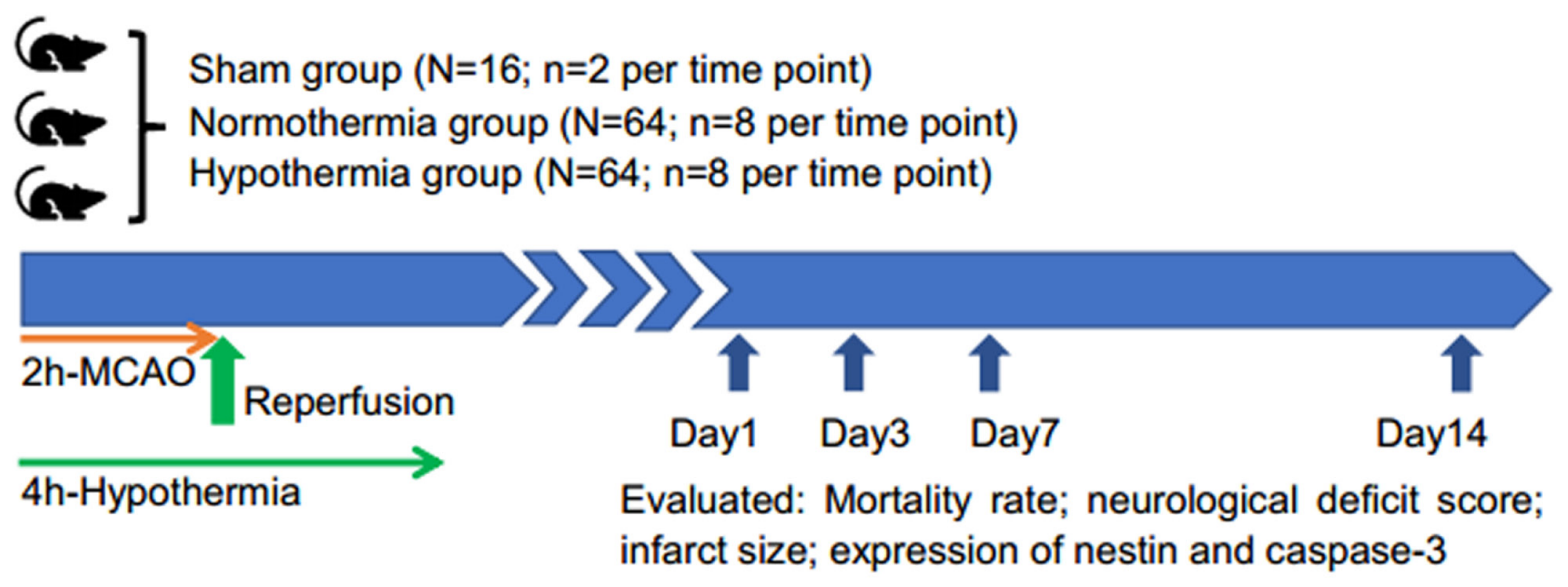
Flow chart of the study

A total of 144 SD rats were randomly distributed into three groups: (1) a sham-operated group under normothermic conditions (sham group, n=16); (2) a MCAO/R group under normothermic conditions (normothermic group, n=64); and (3) a MCAO/R group under hypothermic conditions (hypothermic group, n=64). A flow chart of the study is shown in Figure 5.

### Establishment of a model of ischemic stroke

A modified MCAO/R model was created using an intraluminal suture technique, as described previously [44-45]. The onset of cerebral ischemia was defined as the point at which the MCA was occluded. Following 2 hours of MCAO, the suture was withdrawn to allow reperfusion. The sham group did not experience MCA blockage but other surgical procedures were consistent with the stroke groups.

Rats that died as a direct result of ischemic brain injury, such as significant hemispheric infarction or severe cerebral ischemia, were included in our analysis of survival and mortality, but were excluded from our analysis of neurological deficit score, brain infarct volume, nestin^+^ cells, and caspase-3^+^ cells at given time points.

### MH treatment

MH was induced at the onset of ischemia and maintained for 4 hours as previously described [46]. Briefly, rats in the hypothermic group were placed on an ice blanker machine to cool down their temperatures, and rectal temperature was monitored. The target rectal temperature was set at 33 ± 1°C. All rats reached the target temperature within 15min. After that for 4 hours, the rats were rewarmed slowly to avoid intracranial hypertension [47]. The rectal temperature of the other groups was maintained at a constant 37°C. Rectal temperature was monitored using a digital thermometer (BZT, Beijing, China) with the probe placed approximately 10 mm into the anus.

### Survival and mortality evaluation

All dead rats underwent postmortem examination. Only rats that died as a direct result of ischemic brain injury (i.e., significant hemispheric infarction) were included in our survival and mortality analysis. Survival and mortality were checked daily and we calculated the overall survival rate and total mortality rate for each group.

### The assessment of neurological deficit scores

Neurological deficit score was evaluated for each rat before surgery and on the 1st, 3rd, 7th, and 14th day after the onset of ischemia in each group, and for surviving rats at comparable time points in the sham group. An experienced investigator, who was blinded to the experimental groups, determined neurological deficit score based on a 5-point scale [48].

### Measurement of infarct volume

After neurological evaluation, the 2,3,5-triphenyltetrazolium chloride (TTC; Sigma, St. Louis, USA) staining technique was used to determine the infarct volume of the ischemic brain, as described previously [49]. In brief, to assess infarct size, rats were euthanized by chloral hydrate through the enterocelia on the 1st, 3rd, 7th, and 14th day after the onset of ischemia (n=8 per time point for both normothermic and hypothermia groups; n =2 per time point for the sham group). Then, rat brains were rapidly removed. Coronal sections were cut into five 2-mm slices and stained with standard 2% TTC at 37°C for 10min, followed by overnight immersion in 4% formalin. Finally, images of the brains were captured by camera (Canon SX20, Tokyo, Japan). The infarct areas on each slice were demarcated and analyzed by Image J software (Bethesda, MD, USA). For edema correction, the proportion (%) of the cerebral infarct volume of each rat was determined as 100% × (contralateral hemisphere volume − non-lesioned ipsilateral hemisphere volume) / contralateral hemisphere volume.

### Immunohistochemistry (IHC) staining

To measure nestin^+^ cells, caspase-3^+^ cells in the sub-granular zone of the injured hemisphere, rats were transcardially perfused with 4% paraformaldehyde in 0.1M phosphate buffer (pH=7.3), and decapitated on the 1st, 3rd, 7th, and 14th day after the onset of ischemia (n=8 per time point for the normothermic and hypothermic groups; n=2 per time point for the sham group). Rat brains were removed carefully, then fixed with 10% formalin and embedded in paraffin blocks. The paraffin-embedded brain block was sliced into 7 μm coronal sections at the levels of the dorsal hippocampus (between bregma 2.5 − 4.5 mm) using a freezing microtome (Microm HM525; Thermo Fisher, MA, USA). The sections were routinely deparaffinized and hydrated. After washing with distilled water, the immunohistochemical staining of nestin and caspase-3 was performed, as described previously [50]. The mouse monoclonal anti-nestin antibody (Chemicon, CA, USA) was used to identify neural stem/progenitor cells, and the rabbit polyclonal anti-cleaved caspase-3 antibody (Sigma, MO, USA) was used to identify neuronal apoptosis. Positive immunoreactivity was visualized by treating sections with 0.02% 3,3′-diaminobenzidine (Sigma, St. Louis, USA). Immunostained images were captured with a Nikon camera (Digital Sight DS, Kanagawa, Japan) equipped with Nikon NIS-Elements imaging software (Nikon, Melville, NY, USA) under a light microscope (Olympus, GmbH, Hamburg, Germany). Phosphate-buffered saline was used to replace the antibody in negative control sections and no immunoreactivity was observed in these sections, as expected. Each experiment was repeated three times. The sub-granular zone was defined as a two-cell thick region from the inner margin of the dentate granule cell layer [51]. Three 7-μm sections, spaced 49 μm apart, were selected from each rat, and the number of nestin^+^ cells and caspase-3^+^ cells in the sub-granular zone of the injured hemisphere were counted in each of three non-overlapping high-power fields (three high-power fields × three sections per rat) under magnifications up to 400-fold, and the mean of this data was used for statistical analyses. Each brain was analyzed by one independent investigator who was blinded to the treatment group.

### Statistical analysis

Survival analysis was performed according to the log-rank test while mortality rates were analyzed using the Chi-square (χ2) test. Following testing for homogeneity of variance, quantitative data were expressed as the mean ± standard deviation and analyzed by one-way analysis of variance, followed by Fisher’s least significant difference test to account for multiple comparisons. Correlations between the number of nestin^+^ cells and caspase-3^+^ cells in the sub-granular zone of the injured hemisphere with infarct volume, the neurological deficit score was analyzed by simple linear regression with pooled data. A two-tailed value of p<0.05 was considered to be statistically significant. All statistical analyses were performed using SPSS 20.0 (SPSS, Chicago, IL, USA) and GraphPad Prism 6 (GraphPad, San Diego, CA, USA). All data were analyzed in a blinded manner.

### Compliance with ethical standards

Applicable international, national, and/or institutional guidelines for the ware and use of animals were followed.

## References

[1] Alipanahzadeh H, Soleimani M, Soleimani Asl S, Pourheydar B, Nikkhah A, Mehdizadeh M (2014). Transforming growth factor-α improves memory impairment and neurogenesis following ischemia reperfusion. Cell J.

[2] Subirós N, Pérez-Saad H, Aldana L, Gibson CL, Borgnakke WS, Garcia-Del-Barco D (2016). Neuroprotective effect of epidermal growth factor plus growth hormone-releasing peptide-6 resembles hypothermia in experimental stroke. Neurol Res.

[3] Nagel S, Su Y, Horstmann S, Heiland S, Gardner H, Koziol J, Martinez-Torres FJ, Wagner S (2008). Minocycline and hypothermia for reperfusion injury after focal cerebral ischemia in the rat: effects on BBB breakdown and MMP expression in the acute and subacute phase. Brain Res.

[4] Hoesch RE, Geocadin RG (2007). Therapeutic hypothermia for global and focal ischemic brain injury--a cool way to improve neurologic outcomes. Neurologist.

[5] Liu J, Ji X, Li Z, Zheng H, Zheng W, Jia J, Shen H, Zhang Q, An J (2015). Nestin overexpression promotes the embryonic development of heart and brain through the regulation of cell proliferation. Brain Res.

[6] Matsuda Y, Suzuki G, Kusano T, Kawamoto Y, Yoshimura H, Fuse A, Yokota H, Naito Z, Ishiwata T (2013). Phosphorylation of Thr (1495) of nestin in a mouse model of cerebral ischemia andreperfusion damage. Pathol Int.

[7] Bai Y, Meng Z, Cui M, Zhang X, Chen F, Xiao J, Shen L, Zhang Y (2009). An Ang1-Tie2-PI3K axis in neural progenitor cells initiates survival responses against oxygen and glucose deprivation. Neuroscience.

[8] Luo J, Zheng H, Zhang L, Zhang Q, Li L, Pei Z, Hu X (2017). High-frequency repetitive transcranial magnetic stimulation (rTMS) improves functional recovery by enhancing neurogenesis and activating BDNF/TrkB signaling in ischemic rats. Int J Mol Sci.

[9] Li QQ, Qiao GQ, Ma J, Fan HW, Li YB (2015). Cortical neurogenesis in adult mice after ischemic brain injury: most new neurons fail to mature. Neural Regen Res.

[10] Kwak M, Lim S, Kang E, Furmanski O, Song H, Ryu YK, Mintz CD (2015). Effects of neonatal hypoxic-ischemic injury and hypothermic neuroprotection on neural progenitor cells in the mouse hippocampus. Dev Neurosci.

[11] Xiong M, Li J, Ma SM, Yang Y, Zhou WH (2013). Effects of hypothermia on oligodendrocyte precursor cell proliferation, differentiation and maturation following hypoxia ischemia *in vivo* and *in vitro*. Exp Neurol.

[12] Xiong M, Ma SM, Shao XM, Yang Y, Zhou WH (2012). Hypoxic ischaemic hypothermia promotes neuronal differentiation and inhibits glial differentiation from newly generated cells in the SGZ of the neonatal rat brain. Neurosci Lett.

[13] Xiong M, Cheng GQ, Ma SM, Yang Y, Shao XM, Zhou WH (2011). Post-ischemic hypothermia promotes generation of neural cells and reduces apoptosis by Bcl-2 in the striatum of neonatal rat brain. Neurochem Int.

[14] Yenari MA, Han HS (2013). Influence of therapeutic hypothermia on regeneration after cerebral ischemia. Front Neurol Neurosci.

[15] Silasi G, Klahr AC, Hackett MJ, Auriat AM, Nichol H, Colbourne F (2012). Prolonged therapeutic hypothermia does not adversely impact neuroplasticity after globalischemia in mice. J Cereb Blood Flow Metab.

[16] Silasi G, Colbourne F (2011). Therapeutic hypothermia influences cell genesis and survival in the rat hippocampus following global ischemia. J Cereb Blood Flow Metab.

[17] Kunimatsu T, Kobayashi K, Yamashita A, Yamamoto T, Lee MC (2011). Cerebral reactive oxygen species assessed by electron spin resonance spectroscopy in the initial stage of ischemia-reperfusion are not associated with hypothermic neuroprotection. J Clin Neurosci.

[18] Zhao H, Yenari MA, Cheng D, Sapolsky RM, Steinberg GK (2005). Biphasic cytochrome C release after transient global ischemia and its inhibition byhypothermia. J Cereb Blood Flow Metab.

[19] Siegel CS, McCullough LD (2013). NAD+ and nicotinamide: sex differences in cerebral ischemia. Neuroscience.

[20] Ji Z, Liu K, Cai L, Peng C, Xin R, Gao Z, Zhao E, Rastogi R, Han W, Rafols JA, Geng X, Ding Y (2015). Therapeutic effect of tPA in ischemic stroke is enhanced by its combination with normobaric oxygen and hypothermia or ethanol. Brain Res.

[21] Zgavc T, De Geyter D, Ceulemans AG, Stoop W, Hachimi-Idrissi S, Michotte Y, Sarre S, Kooijman R (2013). Mild hypothermia reduces activated caspase-3 up to 1 week after a focal cerebral ischemia induced by endothelin-1 in mice. Brain Res.

[22] Choi KE, Hall CL, Sun JM, Wei L, Mohamad O, Dix TA, Yu SP (2012). A novel stroke therapy of pharmacologically induced hypothermia after focal cerebral ischemiain mice. FASEB J.

[23] Ntaios G, Dziedzic T, Michel P, Papavasileiou V, Petersson J, Staykov D, Thomas B, Steiner T, European stroke organisation (2015). European Stroke Organisation (ESO) guidelines for the management of temperature in patients with acute ischemic stroke. Int J Stroke.

[24] Nagel S, Papadakis M, Hoyte L, Buchan AM (2008). Therapeutic hypothermia in experimental models of focal and global cerebral ischemia and intracerebral hemorrhage. Expert Rev Neurother.

[25] Muzzi M, Blasi F, Chiarugi A (2013). AMP-dependent hypothermia affords protection from ischemic brain injury. J Cereb Blood Flow Metab.

[26] Maier CM, Sun GH, Kunis D, Yenari MA, Steinberg GK (2001). Delayed induction and long-term effects of mh in a focal model of transient cerebral ischemia: Neurological outcome and infarct size. J Neurosurg.

[27] Cattaneo G, Schumacher M, Wolfertz J, Jost T, Meckel S (2015). Combined selective cerebral hypothermia and mechanical artery recanalization in acute ischemic stroke:*in vitro* study of cooling performance. AJNR Am J Neuroradiol.

[28] Vieites-Prado A, Iglesias-Rey R, Fernández-Susavila H, da Silva-Candal A, Rodríguez-Castro E, Gröhn OH, Wellmann S, Sobrino T, Castillo J, Campos F (2016). Protective effects and magnetic resonance imaging temperature mapping of systemic and focal hypothermia in cerebral ischemia. Stroke.

[29] Cai L, Thibodeau A, Peng C, Ji X, Rastogi R, Xin R, Singh S, Geng X, Rafols JA, Ding Y (2016). Combination therapy of normobaric oxygen with hypothermia or ethanol modulates pyruvate dehydrogenase complex in thromboembolic cerebral ischemia. J Neurosci Res.

[30] Kawai N, Okauchi M, Morisaki K, Nagao S (2000). Effects of delayed intraischemic and postischemic hypothermia on a focal model of transient cerebral ischemiain mice. Stroke.

[31] Karibe H, Chen J, Zarow GJ, Graham SH, Weinstein PR (1994). Delayed induction of mh to reduce infarct volume after temporary middle cerebral artery occlusion in mice. J Neurosurg.

[32] Hu X, Zhang F, Leak RK, Zhang W, Iwai M, Stetler RA, Dai Y, Zhao A, Gao Y, Chen J (2013). Transgenic overproduction of omega-3 polyunsaturated fatty acids provides neuroprotection and enhances endogenous neurogenesis after stroke. Curr Mol Med.

[33] Li YC, Tsai LK, Young TH (2016). Intraventricular infusion of a low fraction of serum enhances neurogenesis and improves recovery in a rodent stroke model. Neurosci Lett.

[34] Sahin S, Alkan T, Temel SG, Tureyen K, Tolunay S, Korfali E (2010). Effects of citicoline used alone and in combination with mild hypothermia on apoptosis induced by focal cerebral ischemia in mice. J Clin Neurosci.

[35] Abeysinghe HC, Bokhari L, Dusting GJ, Roulston CL (2014). Brain remodelling following endothelin-1 induced stroke in conscious mice. PLoS One.

[36] Rueger MA, Muesken S, Walberer M, Jantzen SU, Schnakenburg K, Backes H, Graf R, Neumaier B, Hoehn M, Fink GR, Schroeter M (2012). Effects of minocycline on endogenous neural stem cells after experimental stroke. Neuroscience.

[37] Kawasaki K, Yano K, Sasaki K, Tawara S, Ikegaki I, Satoh S, Ohtsuka Y, Yoshino Y, Kuriyama H, Asano T, Seto M (2009). Correspondence between neurological deficit, cerebral infarct size, and rho-kinase activity in a rat cerebral thrombosis model. J Mol Neurosci.

[38] Wendland MF, Faustino J, West T, Manabat C, Holtzman DM, Vexler ZS (2008). Early diffusion-weighted MRI as a predictor of caspase-3 activation after hypoxic-ischemic insult in neonatal rodents. Stroke.

[39] Rosell A, Cuadrado E, Alvarez-Sabín J, Hernández-Guillamon M, Delgado P, Penalba A, Mendioroz M, Rovira A, Fernández-Cadenas I, Ribó M, Molina CA, Montaner J (2008). Caspase-3 is related to infarct growth after human ischemic stroke. Neurosci Lett.

[40] Westermaier T, Zausinger S, Baethmann A, Steiger HJ, Schmid-Elsaesser R (2000). No additional neuroprotection provided by barbiturate-induced burst suppression under mild hypothermic conditions in mice subjected to reversible focal ischemia. J Neurosurg.

[41] Kuhn HG, Eisch AJ, Spalding K, Peterson DA (2016). Detection and phenotypic characterization of adult neurogenesis. Cold Spring Harb Perspect Biol.

[42] Hu H, Doll DN, Sun J, Lewis SE, Wimsatt JH, Kessler MJ, Simpkins JW, Ren X (2016). Mitochondrial impairment in cerebrovascular endothelial cells is involved in the correlation between body temperature and stroke severity. Aging Dis.

[43] Hooijmans CR, Rovers MM, de Vries RB, Leenaars M, Ritskes-Hoitinga M, Langendam MW (2014). SYRCLE's risk of bias tool for animal studies. BMC Med Res Methodol.

[44] Bai S, Hu Z, Yang Y, Yin Y, Li W, Wu L, Fang M (2016). Anti-inflammatory and neuroprotective effects of Triptolide via the NF-κB signaling pathway in a rat MCAO model. Anat Rec (Hoboken).

[45] Longa EZ, Weinstein PR, Carlson S, Cummins R (1989). Reversible middle cerebral artery occlusion without cranicetomy in mice. Stroke.

[46] Tu Y, Chen C, Sun HT, Cheng SX, Liu XZ, Qu Y, Li XH, Zhang S (2012). Combination of temperature-sensitive stem cells and mh: A new potential therapy for severe traumatic brain injury. J Neurotrauma.

[47] Zhu SZ, Gu Y, Wu Z, Hu YF, Pan SY (2016). Hypothermia followed by rapid rewarming exacerbates ischemia-induced brain injury and augments inflammatory response in mice. Biochem Biophys Res Commun.

[48] Li L, Xiao L, Hou Y, He Q, Zhu J, Li Y, Wu J, Zhao J, Yu S, Zhao Y (2016). Sestrin2 silencing exacerbates cerebral ischemia/reperfusion injury by decreasing mitochondrial biogenesis through the AMPK/PGC-1α pathway in mice. Sci Rep.

[49] Tong L, Cai M, Huang Y, Zhang H, Su B, Li Z, Dong H (2014). Activation of K(2)P channel-TREK1 mediates the neuroprotection induced by sevoflurane preconditioning. Br J Anaesth.

[50] Wei L, Wang J, Cao Y, Ren Q, Zhao L, Li X, Wang J (2015). Hyperbaric oxygenation promotes neural stem cell proliferation and protects the learning and memory ability in neonatal hypoxic-ischemic brain damage. Int J Clin Exp Pathol.

[51] Shetty AK, Hattiangady B, Rao MS, Shuai B (2012). Neurogenesis response of middle-aged hippocampus to acute seizure activity. PLoS One.

